# Perceived Ability to Treat Opioid Use Disorder in West Virginia

**DOI:** 10.13023/jah.0302.04

**Published:** 2021-05-03

**Authors:** A. Brianna Sheppard, Jonathan C. Young, Steve M. Davis, Garrett E. Moran

**Affiliations:** West Virginia University Institute for Community and Rural Health; West Virginia University Health Sciences Center Office of Health Affairs; Department of Health Policy, Management, and Leadership, West Virginia University School of Public Health; Professor, Health Policy, Management, and Leadership, West Virginia University School of Public Health

**Keywords:** Appalachia, medication-assisted treatment, opioid use disorder, stigma, rural health, behavioral health

## Abstract

**Introduction:**

Medication-assisted treatment (MAT) is an evidence-based therapy for opioid use disorder (OUD) that has not been fully implemented in rural areas due to patient, provider, and logistical barriers. Limited information is available on provider perceptions of barriers to MAT in rural Central Appalachia which has very high rates of OUD compared to the rest the United States.

**Purpose:**

Determine perceived barriers for potential prescribers to using MAT, including buprenorphine, as part of treatment for OUD in West Virginia.

**Methods:**

A 30-question, anonymous survey was sent to physicians, physician assistants and advanced practice registered nurses using an online link. Link was distributed through the WV Medicaid provider list, professional association and institutional contact lists, and social media. Comparisons were made by provider waivered or non-waivered status.

**Results:**

Overall, 84% of waivered providers (n = 77) and only 8% of non-waivered providers (n = 341) indicated ever prescribing a form of MAT for OUD; 73% percent of waivered providers were currently prescribing MAT and accepting new patients with OUD. Only 4% of non-waivered providers were currently prescribing MAT and 21% were currently accepting new patients with OUD. Lack of available mental health and psychosocial support services and concerns about diversion or misuse of medication were the top perceived barriers to implementing MAT programs.

**Implications:**

Implementing strategies to improve access to behavioral health care including telehealth and apps, provider training and addressing stigma around OUD treatment were identified as priorities that would help increase providers’ willingness to prescribe medications for OUD treatment.

## INTRODUCTION

Higher rates of occupational injuries and chronic pain coupled with declining economic opportunities and a flood of prescription opioids have fueled rising rates of opioid misuse, resulting in rates of opioid use disorder (OUD) in Central Appalachia that are much higher than other parts of the U.S.^1^ West Virginia currently leads the nation in per capita opioid overdose deaths. It’s rate of drug overdose deaths in 2016 (52 per 100,000) was more than 250% higher than the national rate (19.8 per 100,000).^2^ Medication-assisted treatment (MAT) is an effective, evidence-based therapy for OUD that can reduce cravings and promote relapse prevention that has been associated with a reduction in opioid overdose deaths.^3^ A recent systematic review with meta-analysis observed a 76% reduction in mortality risk among individuals with opioid use disorder while receiving MAT from 16 studies (pooled crude mortality rate [CMR], 0.24 [95% CI, 0.20–0.28]). Individuals not receiving MAT were eight times more likely to die from overdose (RR 8.10, 95% CI, 4.48–14.66) compared to patients receiving treatment.^4^ Despite this demonstrated efficacy, barriers remain to implementation in rural practices. Commonly reported barriers by rural providers include lack of knowledge about MAT; lack of specialty backup; concerns over drug diversion if medications are prescribed; insufficient provider staff time, reimbursement, resource availability including behavioral health, and stigma.^5^,^6^

Efforts are currently underway in West Virginia (WV) to expand buprenorphine treatment via a hub-and-spoke model.^7^ Under a hub-and-spoke model, smaller hospitals and clinics (i.e., spokes) are connected with larger organizations with specialty care (i.e., hubs) to increase local provider education and facilitate local treatment capacity and transfers to specialty care when indicated.^8^ This model has been found to be particularly helpful in the rural setting where declining employment opportunities and other resource strains have led to small hospital and clinic closures.^8^ In particular, the hub and spoke system may overcome the previously observed MAT implementation barriers in rural settings, especially provider lack of knowledge and specialty backup.^8^ However, there is no previously published information regarding perceived MAT prescribing barriers reported by providers in WV, which has the highest opioid overdose death rate in the nation.^9^ A cross-sectional survey of WV providers was conducted to elucidate perceived barriers to implementing MAT programs that may inform policy and other structural interventions to promote diffusion of this life-saving, evidence-based treatment (see Additional Files).

## METHODS

### Participants and Procedures

A cross-sectional study using total population sampling was conducted using an online survey link and recruitment script. The survey link embedded within the recruitment script was sent to the email on file to a total of 20,762 individuals on the WV Medicaid provider list at the time of the study. This list included physicians (N=15,667), physician assistants (N=1833) and advanced practice registered nurses (N=3262). Additional requests to complete the survey using the link sent to the e-mail address on file with WV Medicaid were through professional association and institutional contact lists, and social media.

Participants completed an anonymous 30-item survey adapted from a national study^6^ concerning the ability to use MAT as part of OUD treatment, hosted through Qualtrics (Provo UT). Data were collected between April 2019 and July 2019. MAT was defined as buprenorphine, methadone, naloxone, and injectable naltrexone except where exclusively stated otherwise in the survey. Forced choice, ranking, and open-ended questions were used. The West Virginia University Institutional Review Board acknowledged the study as exempt.

### Data Analysis

Results are presented using descriptive statistics. Data regarding barriers to providing MAT as part of OUD treatment were collected on an ordinal scale and independent samples Mann-Whitney U tests (α=0.05) with Bonferroni corrections to account for multiple comparisons were used to evaluate differences in mean ranks as a function of provider waiver status using SPSS v26. Note that physicians, nurse practitioners, and physician assistants must obtain a waiver from the Drug Enforcement Administration to allow them to prescribe buprenorphine for the treatment of opioid use disorder.^10^ Qualitative analysis of the responses to open-ended questions were reviewed by each member of the research team and consensus was reached on major themes emerging from the data.

## RESULTS

The survey received 780 clicks and the final sample size, based on those who accessed the survey, was 431 respondents who completed at least one question on the survey (55.3%). A total of 303 respondents completed the entire survey (38.8%). Respondents’ age ranged from 25 to 77 years (mean=49 years, n=194).

Sample characteristics are summarized in Table 1. The number of responses to each individual question are provided in Tables 1 and 2.

Major themes that emerged from open comments on reasons providers chose to offer MAT in their clinics they listed better outcomes, number of patients presenting with the problem, lack of providers for pregnant women, patient request, effectiveness, and starting practice in a clinic already providing MAT. Open comments indicated that stigma towards individuals with an OUD and providers who use medication to treat OUD, administrative burden, diversion, and lack of training/comfort in prescribing were perceived barriers to implementing MAT programs. Table 2 illustrates capacity and willingness to prescribe MAT as part of OUD treatment. Providers were most likely to accept patients on their current panel for MAT (n=85), followed by patients in their community not in their practice (n=75), patients of other clinicians in their clinic (n=59) and patients outside their community (n=51). Two questions adapted from published literature^11^ that assessed implicit bias related to treatment of substance use disorder were also included in the survey (see Supplemental Materials) as an additional measure of stigma towards individuals that misuse substances. Respondents reacted more positively to increasing general mental health funding (61%; n=115) versus specific funding for OUD treatment (45%; n=397).

Mean rank was higher for non-waivered providers compared to those with waivers on all perceived barriers to incorporating MAT into treatment of OUD except financial/reimbursement concerns. Lack of available mental health or psychosocial support services and concerns about diversion or misuse of medication were ranked as first and second for all providers, in opposite order for non-waivered and waivered providers, respectively. Statistically significant differences in mean ranks of perceived barriers dependent upon waiver status included: lack of available mental health or psychosocial support services, concerns about diversion or misuse of medication, concerns about legal liability, lack of specialty backup for complex problems, concerns about attracting drug users to practice, outside scope of practice, lack of confidence in ability to manage patients with OUD, and lack of patient need (Table 3).

## IMPLICATIONS

The study goal was to identify the perceived barriers, willingness, and ability to treat OUD in WV, and identify potential targets for policy and procedural changes to support MAT as part of OUD treatment. Only 18% of respondents had completed the training requirements and received their waiver to prescribe buprenorphine as part of OUD treatment. More than half of those who did not currently have a waiver to prescribe buprenorphine indicated a willingness to complete the requirements if free training and compensation were provided. More than 80% of waivered providers indicated they had ever prescribed an OUD MAT drug, higher than the national average of 58%.^12^ Patient need was identified as a reason for prescribing MAT in open-ended comments and ranked lowest on perceived barriers. Results indicate most respondents are currently or are at least willing to incorporate MAT into their practice to address the opioid epidemic in WV.

Lack of available mental health or psychosocial support services and drug misuse/diversion were the two most serious concerns for both waivered and non-waivered providers, the same top barriers identified in the national study from which the current survey was adapted^6^ as well as a similar study of rural primary care providers^5^. Forty-eight of WV’s 55 counties are considered Mental Health Professional Shortage Areas^13^ and a statewide behavioral health workforce learning collaborative was established in 2019 to inform policies and practices addressing this workforce shortage. Work is also underway in WV to expand the use of telehealth and phone-based apps that can support treatment in rural areas with few providers and offer more immediate access to services, including behavioral health. Misuse of diverted buprenorphine is most often done to manage withdrawal symptoms and avoid risk associated with use of street drugs.^14^,^15^ Training on how to identify withdrawal symptoms and adjust dosing accordingly may reduce risk of misuse, abuse, and diversion. A 2016 survey of experienced rural buprenorphine prescribers identified recommendations to avoid problems with the DEA: *all physicians recommended that providers follow all DEA rules strictly (available on the DEA and SAMHSA websites)*,^16^ *keep detailed records, and be prepared for the DEA to visit. Physicians who reported strictly following these rules did not report being concerned about the DEA*.^17^

It is noteworthy that five of the top six concerns were identical for those providers who already held the waiver to prescribe buprenorphine and those who had not yet received the waiver. While there were statistically significant differences between their ratings, as well as differences in rank order, most of the concerns were shared. The very practical concern for financial/payment issues was ranked fourth by those currently waivered but ranked twelfth for those not without a waiver.

Results indicated stigma around provision of MAT for treating OUD. Respondents were more likely to agree reimbursement should be increased for mental health more generally than for OUD specifically, consistent with previous studies exploring implicit bias.^11^ Resistance from practice partners, administration and community were ranked as minor concerns; however, stigma was indicated in open-ended comments such as: *suboxone is a drug of abuse, I strongly believe there should be a better way, substitutes one addiction for another* as well as *attracting drug users to practice*. The latter comment seemed to be a more serious concern for non-waivered providers.

Medication-assisted treatment expansion efforts are being implemented across the state and preliminary findings indicate an increased capacity to address the substance use disorder (SUD)/OUD epidemic, including buprenorphine as part of treatment.^7^ WV’s modified hub and spoke model and Project ECHO provide platforms that can be leveraged to address perceived barriers such as legal liability, incorporating SUD/OUD treatment into scope of practice, increasing confidence in patient management, and addressing stigma.

The WV State Substance Use Response Plan goals for 2020–2022 include increasing the number of providers obtaining waivers by 20% each year, training current and future health care providers on evidence-based approaches to treatment and increasing the number of in-state clinical providers to meet needs of residents seeking SUD treatment.^18^ Data on perceived barriers from the current study can inform priorities and activities at state and regional levels to increase access to SUD treatment.

Limitations include a low response rate and small sample size compared to the number of potential prescribers in WV, limiting generalizability of findings. Two percent of the total population targeted by the survey email responded. Conclusions regarding implicit bias are limited due to the large difference in number of respondents for each question, potential confounds with question order, and less stigmatizing language used in the current study (opioid use disorder) compared to previously published work that used “addiction.”^11^

**SUMMARY BOX**

**What is already known on this topic?**

Medication-assisted treatment (MAT) is an evidence-based therapy for opioid use disorder that has not been fully implemented in rural areas due to patient, provider, and logistical barriers.

**What is added by this report?**

The current study identified perceived barriers of potential prescribers to use MAT including buprenorphine as part of treatment for OUD in West Virginia, a mostly rural state entirely within Appalachia.

**What are the implications for future research?**

These findings can inform efforts to increase access to SUD treatment in the Appalachian region by prioritizing, implementing, and evaluating activities that address providers’ perceived barriers to implementing MAT as part of OUD treatment.

## Figures and Tables

**Table 1:** Sample Characteristics

	N of respondents for each question type	% of respondents for question
**ZIP Code**	259	
West Virginia ZIP code	239	92
**Gender**	264	
Female	144	55
Male	120	46
**Provided clinical practice type**	317	
Rural health clinic	31	10.
Hospital-sponsored clinic	60	19
Community health clinic or federally qualified health center	42	13
Private practice	53	17
Veterans Affairs facility	4	1
Behavioral health facility	24	8
Emergency department	25	8
Inpatient hospital	25	8
Substance use treatment facility	12	4
Other type of facility	41	13
**Type of Reimbursement for MAT (select all that apply)**	203	
Private insurance	149	73
Medicare	147	72
Medicaid	154	76
Self-pay	148	73
WV Public Employees Insurance Agency	139	69

**Table 2:** Capacity and Willingness to Prescribe MAT

Survey Item	Total # of responses	Yes		No	
		n	% Total	n	% Total
Have you taken the mandatory training to allow you to apply for a DEA waiver to prescribe buprenorphine?	428	98	23	330	77
If yes to completing the training, have you received a DEA waiver to allow you to prescribe buprenorphine?	102	77	75	25	25
Have you ever prescribed any form of MAT (buprenorphine, methadone, or naltrexone/Vivitrol) for treatment of OUD?	431	95	22	336	78
Do you currently prescribe MAT for treatment of OUD as part of your practice?	430	69	16	361	84
If no to having a waiver: If free training is made available and you were compensated for your time, would you be willing to obtain your DEA waiver to prescribe buprenorphine?	327	183	56	144	44
Are you currently accepting new patients with OUD?	330	106	32	224	68
Do you feel reimbursement should be increased for treatment of OUD?	397	179	45	218	55
Do you feel reimbursement should be increased for treating mental illness?	115	70	61	45	39

**Table 3:** Mean Ranking of Barriers to Implementing MAT

WAIVERED			Likert Scale: 1–Not a concern 2–Minor concern 3–Serious concern 4–Very serious concern	NOT WAIVERED			Mann-Whitney		
N = 77				N = 319*			Bonferroni Correction α=0.003		
n	Rank	Mean	Barrier	n	Rank	Mean	N	U	p
70	2	2.51	Lack of available mental health or psychosocial support services	252	1	3.16	322	6100	**<0.0001**
70	1	2.52	Concerns about diversion or misuse of medication	253	2	2.96	323	6782	**0.002**
69	5	2.29	Concerns about legal liability	254	3	2.82	323	6437	**0.0004**
69	6	2.24	Lack of specialty backup for complex problems	253	4	2.71	322	6467	**0.0001**
68	3	2.42	Time constraints	254	6	2.54	322	8146	0.455
70	11	1.84	Concerns about attracting drug users to your practice	253	7	2.51	323	6112	**<0.0001**
70	13	1.45	Outside my scope of practice	258	5	2.60	328	4042	**<0.0001**
70	14	1.41	Lack of confidence in my ability to manage patients with OUD	254	8	2.38	324	4313	**<0.0001**
70	8	2.01	Concerns with DEA intrusion on your practice	251	9	2.18	321	8338	0.495
70	4	2.39	Financial/reimbursement concerns	253	12	1.99	323	10713	0.005
70	10	1.88	Resistance from practice partners	253	10	2.06	323	8232	0.342
69	12	1.72	Resistance from practice administration	250	11	2.03	319	7557	0.094
70	7	2.07	Resistance from community	253	13	1.93	323	9705	0.193
50	9	1.98	Other	126	14	1.86	176	3181	0.907
68	15	1.31	Lack of patient need	254	15	1.67	322	6915	**0.0026**

* Reflects the number of respondents out of 330 total respondents indicating they had not completed the mandatory training to allow for application for a DEA waiver to prescribe buprenorphine that provided a response to questions identifying barriers.

Individual ns indicate the number of providers that responded “Yes” to each barrier listed.
